# Cardiometabolic Risks of Blonanserin and Perospirone in the Management of Schizophrenia: A Systematic Review and Meta-Analysis of Randomized Controlled Trials

**DOI:** 10.1371/journal.pone.0088049

**Published:** 2014-02-04

**Authors:** Taro Kishi, Yuki Matsuda, Nakao Iwata

**Affiliations:** Department of Psychiatry, Fujita Health University School of Medicine, Toyoake, Aichi, Japan; Chiba University Center for Forensic Mental Health, Japan

## Abstract

**Background:**

The present study aimed to evaluate cardiometabolic risks [weight gain, blood lipid levels (total cholesterol and triglycerides), blood glucose levels, hemoglobin A_1c_ (HbA_1c_) levels, and corrected QT interval (QTc) prolongation] associated with the use of blonanserin and perospirone versus other antipsychotics in the management of patients with schizophrenia.

**Method:**

We conducted a systematic review and meta-analysis of patient data from randomized controlled trials comparing blonanserin or perospirone with other antipsychotics.

**Results:**

In total, 4 blonanserin studies (n  = 1080) were identified [vs. risperidone (2 studies, n = 508); vs. haloperidol (2 studies, n = 572)]. Blonanserin produced less weight gain compared with risperidone (weighted mean difference = −0.86, 95% confidence intervals = −1.36 to −0.36, p = 0.0008; 2 studies, 480 patients). However, no significant differences were observed in blood lipid, glucose, and HbA_1c_ levels or QTc prolongation between blonanserin and risperidone or haloperidol. For perospirone studies, 5 studies [562 adult patients with schizophrenia randomized to perospirone (n = 256), olanzapine (n = 20), quetiapine (n = 28), risperidone (n = 53), aripiprazole (n = 49), haloperidol (n = 75), or mosapramine (n = 81)] were identified. Perospirone did not differ from other antipsychotics with regard to weight gain and total cholesterol levels.

**Conclusions:**

Our results suggest that blonanserin is associated with a lower of weight gain compared with other antipsychotics. Because the number of studies was small, additional controlled clinical trials with larger number of patients are indicated.

## Introduction

Second-generation or atypical antipsychotics (SGAs) have been reported to be more efficacious (particularly for negative symptoms) and associated with a lower risk of extrapyramidal symptoms and hyperprolactinemia compared with first-generation or typical antipsychotics (FGAs) [1]. In fact, a meta-analysis of schizophrenia treatment studies revealed that clozapine, olanzapine, and risperidone were superior to pooled FGAs in the improvement of overall symptoms [clozapine: standardized mean difference (SMD) = −0.52, 95% confidence interval (CI) = −0.75 to −0.29; olanzapine: SMD = −0.28, 95% CI = −0.38 to −0.18; risperidone: SMD = −0.13, 95% CI = −0.22 to 0.05] [1]. However, SGAs were reported to have higher metabolic risks, including weight gain, compared with FGAs [1]. Moreover, another meta-analysis of schizophrenia treatment studies reported that there were the differences in efficacy and safety, including metabolic risks and extrapyramidal symptoms, among SGAs [2]–[4]. For example, olanzapine was superior to aripiprazole (SMD = −0.22, 95% CI = −0.33 to −0.12) and sertindole (SMD = −0.23, 95% CI = −0.43 to −0.03) in overall efficacy [2]. However, olanzapine and clozapine have been demonstrated to have a higher risk of metabolic abnormalities compared with other SGAs [3]. For example, olanzapine revealed a higher risk of weight gain compared with amisulpride (SMD  = 0.52, 95% CI  = 0.19 to 0.85), aripiprazole (SMD  = 0.63, 95% CI  = 0.34 to 0.92), asenapine (SMD  = 0.43, 95% CI  = 0.30 to 0.56), lurasidone (SMD  = 0.97, 95% CI  = 0.74 to 1.20), paliperidone (SMD  = 0.25, 95% CI  = 0.12 to 0.39) and risperidone (SMD  = 0.43, 95% CI  = 0.26 to 0.60) [3]. On the other hand, risperidone was associated with the need for more use of antiparkinson medication compared with clozapine [relative risk (RR)  = 2.57, number needed to harm (NNH)  = 6], olanzapine (RR  = 1.28, NNH  = 17), quetiapine (RR  = 1.98, NNH  = 20), and ziprasidone (RR  = 1.42, NNH  = 17) [4]. When comparing antipsychotics (regarding overall symptoms as efficacy and weight gain as safety), because the effect size of efficacy is smaller than that of safety such as weight gain, several guidelines for the management of schizophrenia have recommended that the safer antipsychotic should be used for patients with schizophrenia.

Recently, we published 2 meta-analyses of schizophrenia; 1 of them was a meta-analysis of blonanserin regarding efficacy and safety in schizophrenia [5]. The article reported following 2 primary results: (1) although blonanserin exhibits similar effects as risperidone and haloperidol with respect to the overall psychopathology of schizophrenia, blonanserin has more beneficial effects on negative symptoms compared with haloperidol; (2) blonanserin has a lower risk of hyperprolactinemia compared with other pooled antipsychotics. Although dizziness and akathisia occur significantly less often with blonanserin than with haloperidol, blonanserin has a higher risk of akathisia than risperidone. The second article was a meta-analysis regarding efficacy and safety of perospirone in schizophrenia [6]; the article reported following 3 primary results: (1) although perospirone did not differ from other pooled antipsychotics regarding the reduction in Positive and Negative Syndrome Scale (PANSS) negative and general subscale scores or regarding discontinuation because of any cause, inefficacy, and side effects, perospirone was inferior to other pooled antipsychotics in the reduction of PANSS total scores and positive subscale scores; (2) perospirone was superior to haloperidol in the reduction of PANSS negative subscale scores; (3) perospirone had lower scores related to extrapyramidal symptoms compared with other pooled antipsychotics. However, because we did not acquire available data with regard to cardiometabolic risks for meta-analyses in our previous articles, we did not perform meta-analyses addressing cardiometabolic risks of blonanserin and perospirone in patients with schizophrenia in our previous articles [5], [6]. The present study aimed to evaluate cardiometabolic risks [weight gain, blood lipid levels (total cholesterol and triglycerides), blood glucose levels, hemoglobin A1c (HbA1c) levels, and corrected QT interval (QTc) prolongation] associated with the use of blonanserin and perospirone versus other antipsychotics in the management of patients with schizophrenia.

## Materials and Methods

### Inclusion Criteria and Search Strategy, Data Extraction, and Outcomes

We selected only randomized controlled trials (RCTs) using blonanserin or perospirone therapy in patients with schizophrenia. This meta-analysis was performed according to guidelines of preferred reporting items for systematic reviews and meta-analyses (PRISMA, 2009) [7]. We performed a systematic literature review according to the PICO strategy (Patients: schizophrenia, Intervention: blonanserin or perospirone, Comparator: other antipsychotics, and Outcome: cardiometabolic risks). We included open-label and double-blinded RCTs comparing blonanserin or perospirone with other antipsychotics for patients with schizophrenia or schizophrenia-like psychoses. We allowed inclusion of nondouble-blinded studies to include more studies in the meta-analysis. To identify relevant studies, we searched PubMed, Cochrane Library databases, Google Scholar, and PsycINFO citations (without language restrictions) published up to January 2, 2013, using following key words: “randomized,” “random,” or “randomly” and “blonanserin” or “perospirone” and “schizophrenia.” Additional eligible studies were also sought by a hand search of reference lists from primary articles and relevant reviews. Three authors (T.K., Y.M., and N.I.) assessed inclusion and exclusion criteria for each identified study. Discrepancies in different coding forms were resolved by discussions between authors (T.K., Y.M., and N.I.). Three authors (T.K., Y.M., and N.I.) independently extracted, assessed, and entered data into the Review Manager. When data required for the meta-analysis were missing, first/corresponding authors were contacted for additional information (including endpoint scores). Unpublished data were provided for this meta-analysis by Dr. Jaewon Yang, Dr. Seung-Hyun Kim, Dr. Sadanori Miura, Dr. Mitsukuni Murasaki, Dr. Yoshiteru Takekita, Bukwang Pharm. Co., Ltd., and Dainippon Sumitomo Pharma Co., Ltd. Moreover, we assessed the methodological quality of trials using Cochrane risk-of-bias criteria (http://bmg.cochrane.org/assessing-risk-bias-included-studies).

### Outcomes

The primary outcome was weight gain. Secondary outcomes were changes in blood lipid levels (total cholesterol and triglycerides), blood glucose levels, HbA_1c_ levels, and QTc prolongation (Bazett method) on electrocardiography.

### Statistical Analysis

We pooled data for side effects. We based our analysis on intention-to-treat (ITT) or modified ITT data (i.e., at least 1 dose or at least 1 follow-up assessment); no observed case data were included. The meta-analysis was performed using the Review Manager (RevMan), version 5.1, for Windows (http://ims.cochrane.org/revman). To combine studies, we used random effects model by DerSimonian and Laird [8]. We used this conservative model to address the possibility that underlying effects that differ across studies and populations would be heterogeneous. We used weighted mean difference (WMD) and 95% CIs for continuous data. In cases where I-squared values were >50%, sensitivity analyses were conducted to seek reasons for the heterogeneity [9]. However, we could not determine any significant heterogeneity in all outcomes between both treatment groups. Finally, funnel plots were visually inspected to explore the possibility of publication bias.

## Results

### Blonanserin

#### Study characteristics

**Figure 1:**
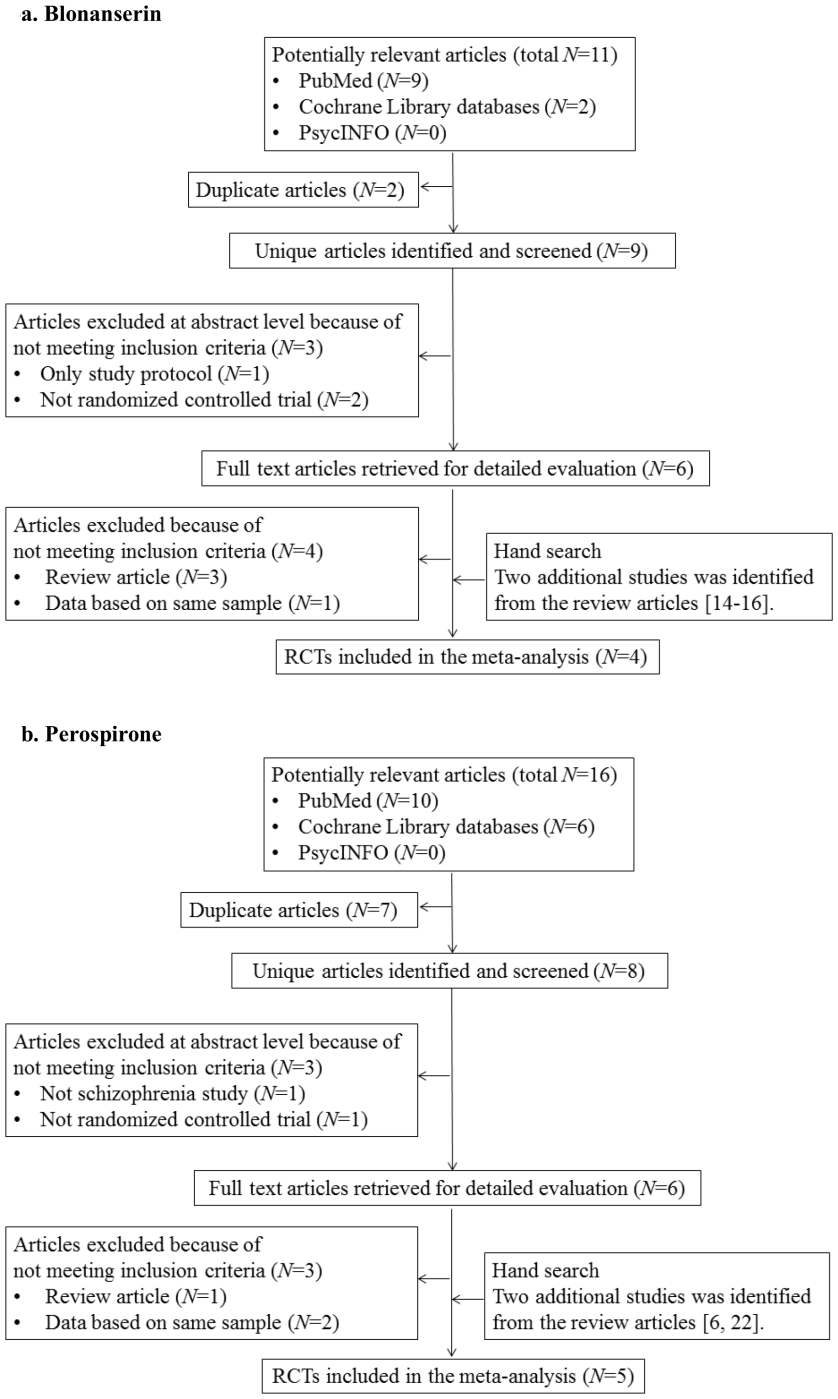
PRISMA flow diagram. 1-a. Blonanserin. 1-b. Perospirone.

The PubMed search yielded 11 studies using abovementioned key words for screening (**FIGURE** **1** **-a**). We excluded 2 duplicate studies across 3 databases as well as 3 studies based on the title or abstract review. Additional 4 full-text articles were excluded because they were either review papers (N  = 3) or data were based on the same sample (N  = 1), yielding 2 eligible studies [10], [11] (**FIGURE** **1** **-a**). In addition, 2 other studies [12], were identified from the review [14]–[16]. In total, we identified 4 studies involving 1080 patients with schizophrenia that reported data from double-blinded, randomized controlled trials of blonanserin for schizophrenia, thus meeting our inclusion criteria (vs. risperidone: n  = 508; vs. haloperidol: n  = 572). The mean study duration was 7.5 weeks; 3 trials lasted 8 weeks, and 1 trial lasted 6 weeks. Sample sizes ranged from 206 to 307 patients. The mean age of the study population was 41.0 years. Two studies [11], [13] used the SGA risperidone, and the other 2 [10], [12] used the FGA haloperidol as a comparator. All studies were sponsored by the pharmaceutical industry. Two studies were published in English and the other 2 in Japanese. Two studies were conducted in Japan, 1 in Korea, and the remaining 1 was conducted in the United States and Europe. All studies were of high methodological quality based on Cochrane Risk of Bias Criteria because all blonanserin studies were double-blinded RCTs and mentioned required details of the study design (**FIGURE S1 and S2**). Characteristics of studies included have been presented in our previous paper [16].

#### Cardiometabolic risks

**Figure 2:**
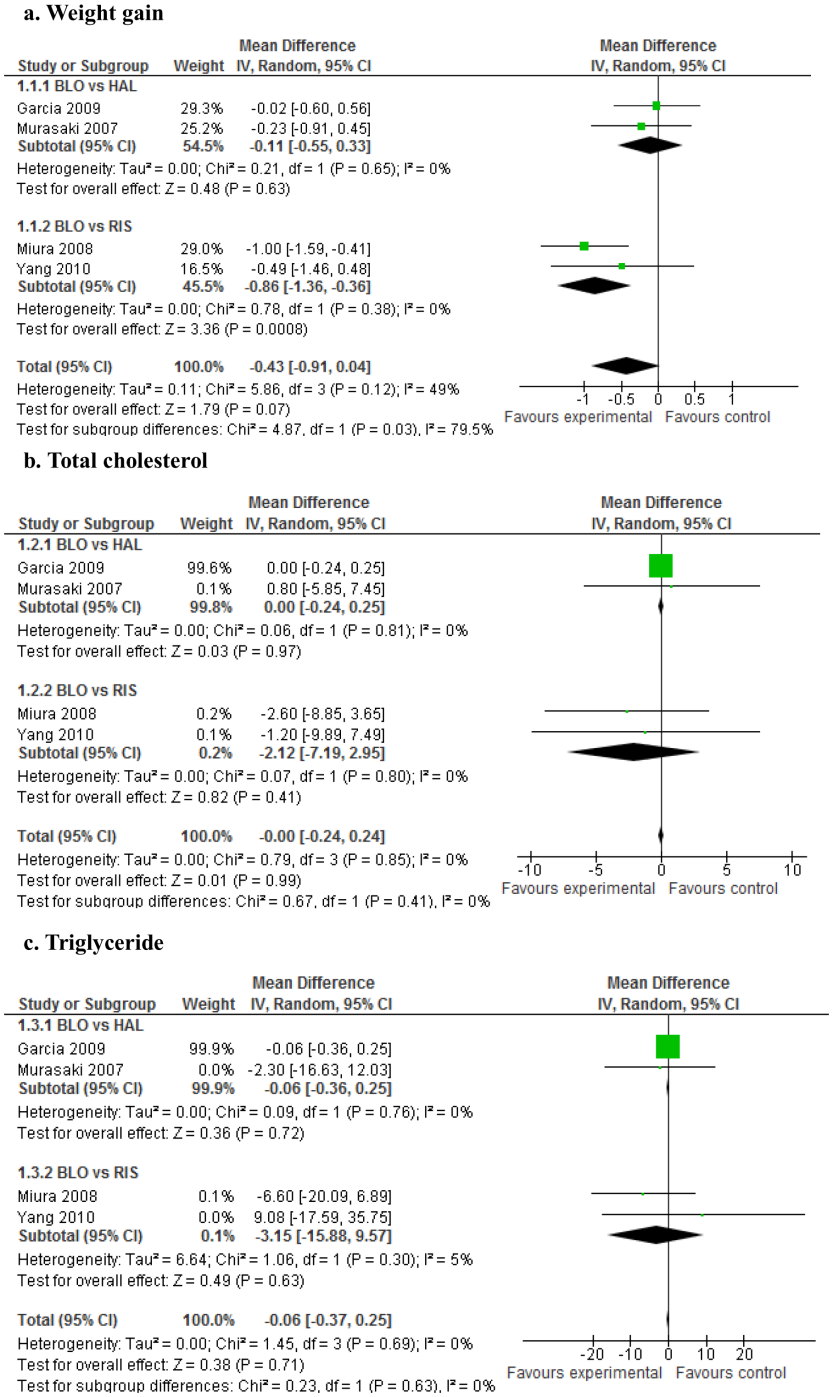
Forest plot: blonanserin (weight gain and total cholesterol and triglyceride levels). 2-a. Weight gain. 2-b. Total cholesterol. 2-c. Triglyceride. BLO: blonanserin, HAL: haloperidol, RIS: risperidone.

Comparing blonanserin with other pooled antipsychotics, no significant differences were observed in weight gain, blood lipid levels (total cholesterol and triglycerides), blood glucose levels, HbA_1c_ levels, or QTc prolongation (**FIGURE 2** **and** **3**). However, blonanserin apparently produced less weight gain compared with risperidone (WMD = −0.86, 95% CI = −1.36 to −0.36, p  = 0.0008; 2 studies, 480 patients; **FIGURE 2** **-a**). Visual inspection of the funnel plot for weight change in blonanserin vs. other antipsychotics did not suggest the presence of publication bias, but the sensitivity was limited by the small number of 4 studies.

**Figure 3:**
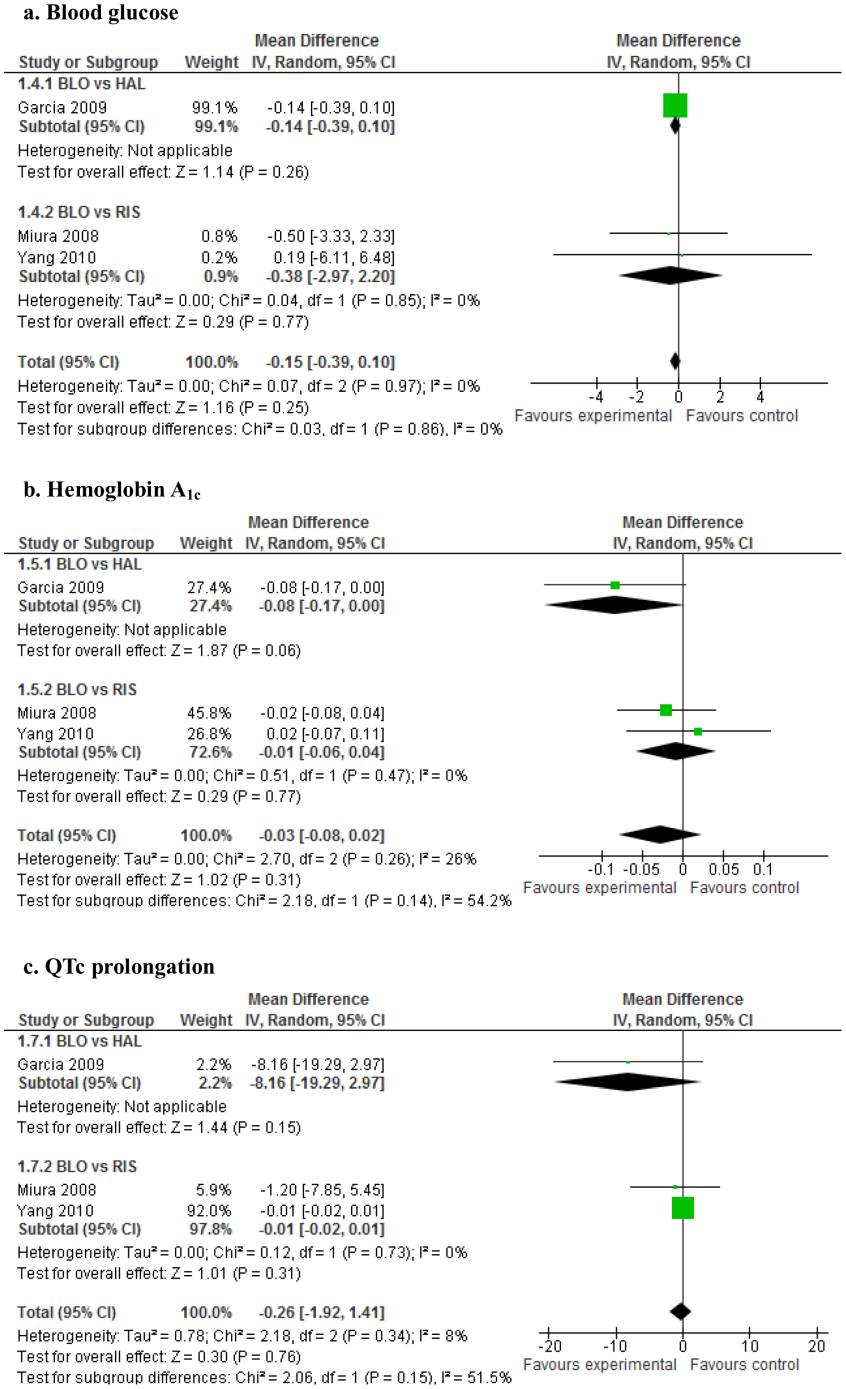
Forest plot: blonanserin (blood glucose and hemoglobin A_1c_ levels and QTc prolongation). 3-a. Blood glucose. 3-b. Hemoglobin A_1c._ 3-c. QTc prolongation. BLO: blonanserin, HAL: haloperidol, RIS: risperidone.

### Perospirone

#### Study characteristics

The search in PubMed, Cochrane Library databases, Google Scholar, and PsycINFO yielded 11 results (**FIGURE 1** **-b**). We excluded 2 duplicate studies across 3 databases as well as 3 studies based on the title or abstract review. An additional 3 full-text articles were excluded because they were either review papers (N  = 1) or data were based on the same sample (N  = 2), yielding 3 eligible studies [17]–[19] (**FIGURE 1** **-b**). Finally, 2 additional studies [20], [21] were identified from the review articles [6], [22]. Across 5 RCTs (mean duration: 9.6 weeks; range: 4–12 weeks), 562 adult patients with schizophrenia were randomized to perospirone (n  = 256), olanzapine (n  = 20) [17], quetiapine (n  = 28) [17], risperidone (n  = 53) [17], [19], aripiprazole (n  = 49) [18], haloperidol (n  = 75) [21], or mosapramine (n  = 81) [20]. Sample sizes ranged from 66 to 159 participants. Three of 5 studies were published in English [17], [19], and other 2 studies [20], [21] were published in Japanese. Three of 5 studies were sponsored by the pharmaceutical industry [19]–[21]. Two of 5 studies [20], [21] were of high methodological quality based on Cochrane Risk of Bias Criteria because these 2 studies were double-blinded RCTs and mentioned required details of the study design (**FIGURE S1 and** S**2**). However, other 3 studies [17]–[19] were open-label RCTs. Characteristics of studies have been presented in our previous paper.

#### Cardiometabolic risks

Perospirone did not differ from other antipsychotics with regard to weight gain and total cholesterol. Because there were no available data regarding other cardiometabolic outcomes, we did not conduct that meta-analysis (**FIGURE S3**). Visual inspection of the funnel plot for weight gain in perospirone vs. other antipsychotics did not suggest the presence of publication bias, but the sensitivity was limited by the small number of 5 studies.

## Discussion

To our knowledge, this is the first comprehensive meta-analysis of cardiometabolic risks of blonanserin and perospirone for patients with schizophrenia. Although previous studies did not report that blonanserin was associated with a lower risk of weight gain, trends in this direction have been reported. Our meta-analysis enabled us to obtain greater statistical power compared with that in past studies (Cochrane Handbook for Systematic Reviews of Interventions, http://handbook.cochrane.org/); therefore, we were able to establish that blonanserin had a lower risk of weight gain compared with risperidone. Antipsychotic affinity for the histamine H_1_ receptor is most closely linked to increased weight gain, although affinity for dopamine D_2_, serotonin _1A_, serotonin _2C_ and α_2_-adrenergic receptors may also be involved [23]–[25] . Blonanserin lacks significant affinity for histamine H_1,_ serotonin _1A_, α_2_-adrenergic receptors [16] . Haloperidol also lacks significant affinity for histamine H_1_, serotonin _1A_, serotonin_ 2C_ and α_2_-adrenergic receptors [16] . On the other hand, risperidone has a high affinity for histamine H_1_, dopamine D_2_, serotonin _2C_ and α_2_-adrenergic receptors [16] . Olanzapine and clozapine also have a high affinity for histamine H_1_ and serotonin _2C_ receptors [16] . These mechanisms may be involved in the different effects seen among the antipsychotic groups. Although several studies have reported a difference in the degree of weight gain induced by antipsychotics between patients with antipsychotic-naïve schizophrenia and those with chronic schizophrenia (i.e., patients with antipsychotic-naïve schizophrenia have revealed more weight gain than those with chronic schizophrenia), and although some have revealed a varying degree of weight gain, depending on the antipsychotic dosage (e.g., risperidone dosage was associated with an increased degree of weight gain) [25], [26], our results indicate that blonanserin may be the safest antipsychotic with regard to weight gain. However, because the number of studies was small, additional controlled clinical trials with larger number of patients with antipsychotic-naïve schizophrenia are indicated. There are other limitations to our findings. Although blonanserin was significantly associated with a lower weight gain risk compared with other antipsychotics, the significant result detected may be driven by the Miura study data [13] . Moreover, although no obvious publication bias could be identified, the possibility of the bias would be excluded from the significant result based on a subgroup analysis with only two studies.

### Conclusion

Our results suggest that blonanserin is associated with a lower weight gain risk compared with other antipsychotics. However, because this meta-analysis includes only 4 blonanserin RCTs and 5 perospirone RCTs, future research should generate more safety data using larger samples.

## Supporting Information

Figure S1 **Risk of bias graph.**
(TIF)

Figure S2 **Risk of bias summary.**
(TIF)

Figure S3 **Forest plot: perospirone (weight gain and total cholesterol levels). 3-a. Weight gain 3-b. Total cholesterol.** ARI: aripiprazole, HAL: haloperidol, MOS: mosapramine, PER: perospirone, RIS: risperidone.
(TIF)

Checklist S1 **PRISMA 2009 Checklist.**
(DOC)
