# Supplementary figures and images for: Cardiometabolic Risks of Blonanserin and Perospirone in the Management of Schizophrenia: A Systematic Review and Meta-Analysis of Randomized Controlled Trials
Source: PLoS One. 2014 Feb 4;9(2):e88049. doi: 10.1371/journal.pone.0088049 (PMC3913743; doi:10.1371/journal.pone.0088049)

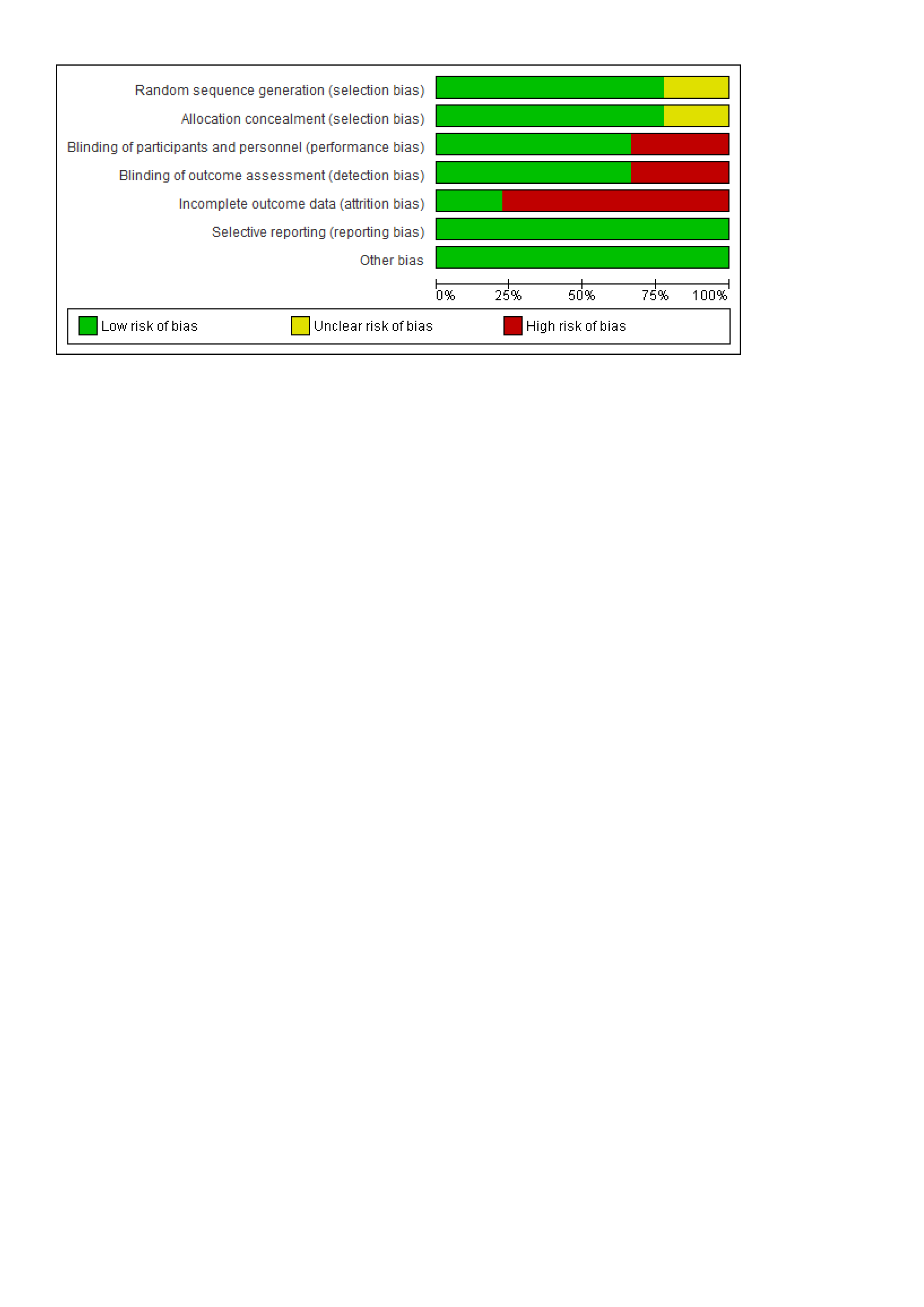

Supplement: Figure S1 — Risk of bias graph. (TIF) [file pone.0088049.s001.tif]

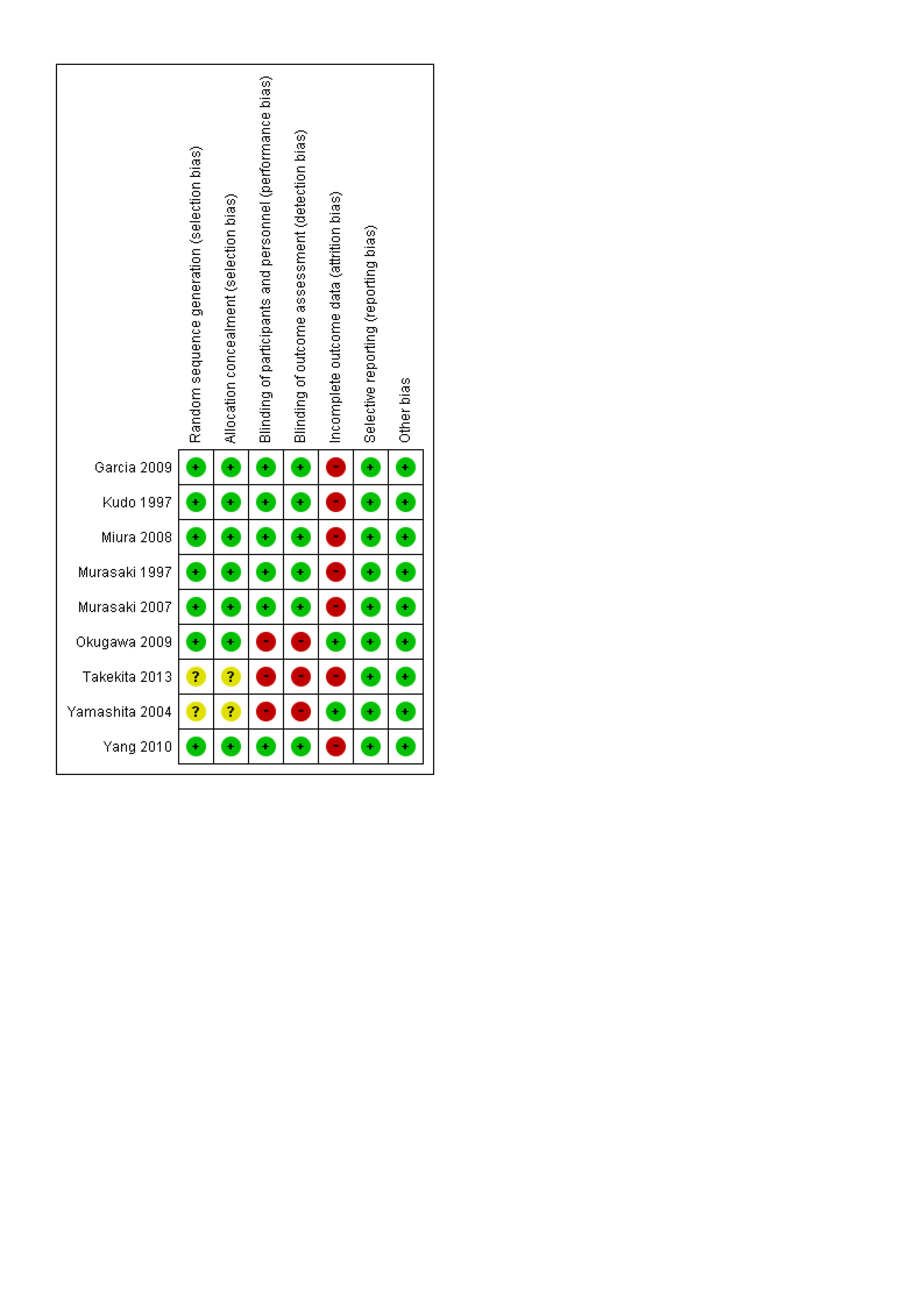

Supplement: Figure S2 — Risk of bias summary. (TIF) [file pone.0088049.s002.tif]

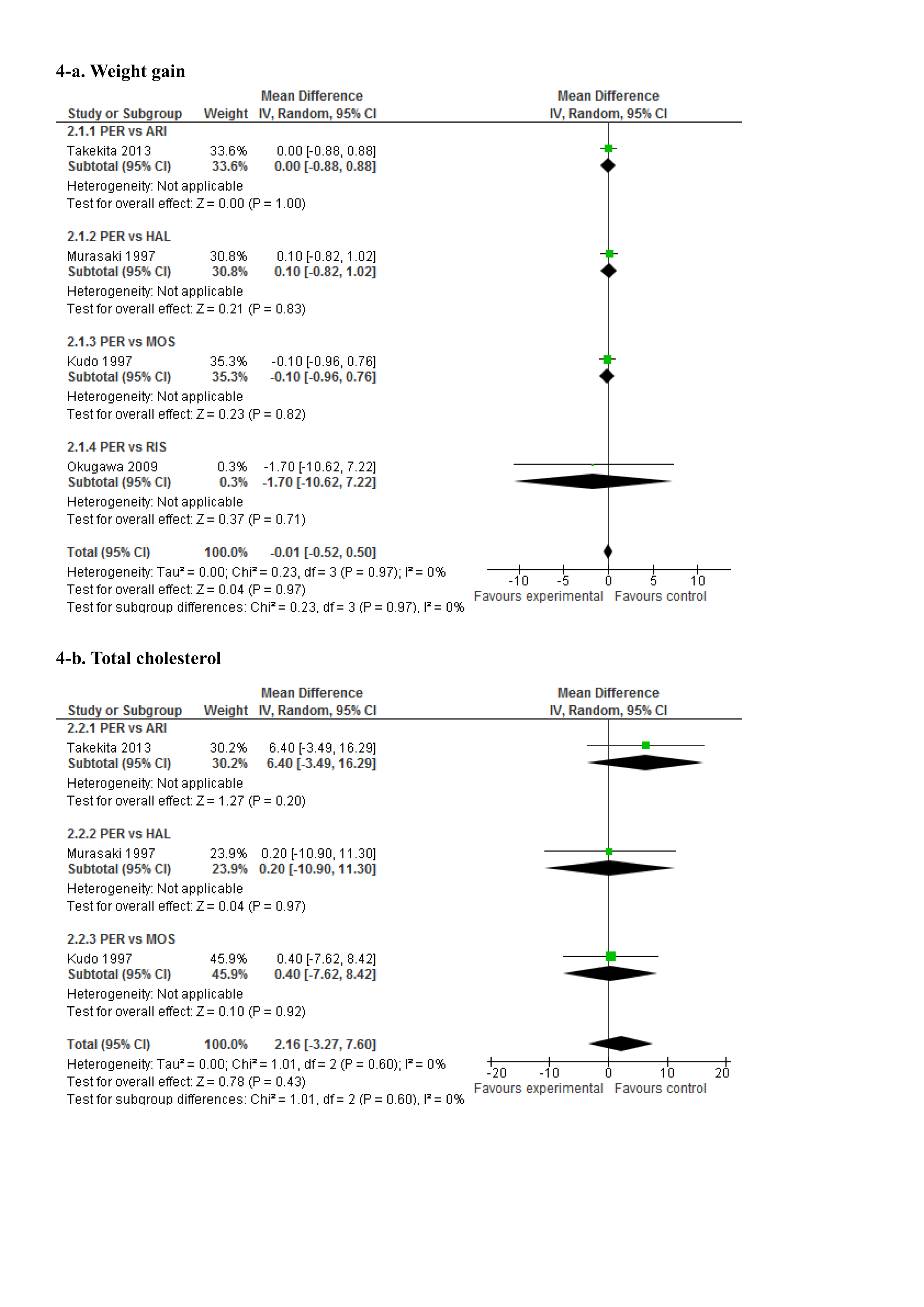

Supplement: Figure S3 — Forest plot: perospirone (weight gain and total cholesterol levels). 3-a. Weight gain 3-b. Total cholesterol. ARI: aripiprazole, HAL: haloperidol, MOS: mosapramine, PER: perospirone, RIS: risperidone. (TIF) [file pone.0088049.s003.tif]
